# The clinical significance of D-dimer concentrations predicting the risk of venous thromboembolism in patients with hyperemesis gravidarum

**DOI:** 10.1590/1806-9282.20250088

**Published:** 2025-08-08

**Authors:** Berrin Göktug Kadıoglu, Ayse Nur Aksoy, Selvihan Tapanoglu Karaca, Konca Altınkaynak, Nurcan Yoruk

**Affiliations:** 1 University of Health Sciences, Erzurum City Hospital, Department of Obstetrics and Gynecology – Erzurum, Türkiye.; 2 University of Health Sciences, Erzurum City Hospital, Department of Biochemistry – Erzurum, Türkiye.

**Keywords:** D-dimer, Fibrinogen, Hyperemesis, Pregnancy, Venous thromboembolism

## Abstract

**OBJECTIVE:**

The aim of this prospective cross-sectional study was to determine serum D-dimer and fibrinogen concentrations in pregnant women with hyperemesis gravidarum and compare them with those of healthy pregnant women.

**METHODS:**

The study group consisted of 50 patients diagnosed with hyperemesis gravidarum. The control group consisted of 50 healthy pregnant women. Maternal characteristics such as age, gestational age, body mass index, gravida, and parity were recorded. Following a 12-h fasting period, venous blood samples of 10 cc were collected. The blood samples were centrifuged, and serum was stored at −80°C until assay. Blood count, coagulation profiles, and biochemical analyses were performed, and the results were analyzed using appropriate statistical tests.

**RESULTS:**

The data of 45 patients in the hyperemesis gravidarum group and 47 patients in the control group were analyzed. Serum levels of hemoglobin, white blood cell and platelet counts, prothrombin time, activated partial thromboplastin time, fibrinogen, D-dimer, and international normalized ratio were similar between groups (p>0.05). There was a positive association between serum D-dimer levels and serum fibrinogen levels (p=0.039, r=0.215). Also, a positive association was observed between serum D-dimer levels with the degree of ketonuria (p=0.008, r=0.274) and the number of vomitings per day (p=0.02, r=0.346) in hyperemesis gravidarum patients.

**CONCLUSION:**

Serum D-dimer levels were not different in patients with hyperemesis gravidarum versus healthy women during the first trimester of pregnancy. Pregnant women tend to have higher D-dimer concentrations as hyperemesis gravidarum severity increases. Serum D-dimer levels cannot be used for the evaluation of potential venous thromboembolism in patients diagnosed with hyperemesis gravidarum.

## INTRODUCTION

Hyperemesis gravidarum (HEG) is a severe form of nausea and vomiting during pregnancy, characterized by ketonuria, electrolyte imbalance, dehydration, acid–base disturbances, and weight loss. It affects approximately 0.5–2% of all pregnancies^1,2^. Existing studies have not fully clarified the pathogenesis and etiology of HEG. Uçkan et al.^3^ reported increased serum ischemia-modified albumin levels and decreased total sulfhydryl levels in HEG patients compared to healthy pregnant women. Similarly, Şimşek et al.^4^ reported higher serum total oxidative status and lower total antioxidant status levels in women with HEG compared to healthy pregnancies.

D-dimer is a fibrin degradation product formed after coagulation system activation. Studies on the clinical value of elevated D-dimer levels in predicting potential venous thromboembolism (VTE) have conflicting results. In a recent study, Richardson et al.^5^ analyzed 995 patients aged 60 years and above who were admitted for an acute medical illness. They reported elevated D-dimer levels on admission in these patients, and VTE was observed in only 1.8% of them. They concluded that D-dimer levels should not be used for the routine evaluation of potential VTE. However, Karny-Epstein et al.^6^ reported that D-dimer may be used in hospitalized patients to exclude VTE, with a sensitivity of almost 80%. On the other hand, D-dimer levels naturally rise during pregnancy, and factors such as obesity, advanced maternal age, and comorbidities like diabetes can exacerbate this increase^7^. A study investigating reference ranges for D-dimer and fibrinogen during pregnancy reported values between 167 and 721 ng/mL for the first trimester^8^.

In severe cases of HEG, excessive nausea and vomiting lead to dehydration and electrolyte imbalance, which requires hospitalization^9^. Medical problems requiring hospitalization, such as trauma, infections, inflammation, or chronic diseases and prolonged immobilization caused by hospitalization, have been shown to elevate the risk of thrombosis and increase serum D-dimer levels^10,11^. Also, HEG adversely affects the daily activities of pregnant women and may lead to anxiety and depression^12,13^. Keskinkılıc et al.^14^ reported two cases of fatal pulmonary thromboembolism in first-trimester HEG pregnancies.

We hypothesized that serum D-dimer values could be used as a marker to predict the occurrence of VTE in patients with HEG and to initiate prophylactic anticoagulant therapy in these patients. There are no studies in the literature investigating serum D-dimer levels in patients diagnosed with HEG. Therefore, the main goal of this research was to determine serum D-dimer and fibrinogen concentrations in pregnant women with HEG and compare them with the values of healthy pregnant women. Also, the relationship between serum D-dimer levels and other biochemical parameters was investigated.

## METHODS

This prospective cross-sectional study was approved by the Ethical Committee of Medical Faculty, Ataturk University (project number: B.30.2.ATA.0.01.00/227) and was conducted at the Gynecology and Obstetrics Clinic of Erzurum City Hospital between May 2024 and July 2024. This study was conducted in accordance with the Declaration of Helsinki, and written informed consent was obtained from all participants.

The study group consisted of 50 patients diagnosed with HEG aged between 18 and 40 years, at 6–12 weeks of gestation. The HEG was defined as severe vomiting (≥3 episodes/day), positive ketonuria, and weight loss >5% of pre-pregnancy weight. The control group consisted of 50 healthy pregnant women without nausea and vomiting matched in demographic characteristics, including maternal age, gestational age, and body mass index (BMI). Patients with BMI≥30 kg/m^2^, smoking, alcohol consumption, polycystic ovaries, irregular menstrual cycles, chronic diseases such as diabetes and hypertension, thyroid disease, multiple gestation, gestational trophoblastic disease, vaginal bleeding, and gastrointestinal disorders were excluded. Patients conceived by assisted reproductive technologies and receiving anticoagulant therapy were also excluded. All participants were asked about the first day of their last menstrual period, and the gestational week was verified through ultrasonography. Maternal characteristics such as age, gestational age, BMI, gravida, and parity were recorded. BMI was calculated as weight in kilograms divided by the square of height in meters (kg/m^2^). Following a 12-h fasting period, venous blood samples of 10 cc were collected from the antecubital vein into tubes without anticoagulants in the early morning. The blood samples were centrifuged at 3,500×g for 15 min, and the separated serum was stored at −80°C until the assay. Blood count, coagulation profile, and biochemical analyses were performed, and the results were recorded. Coagulation markers included D-dimer, fibrinogen, international normalized ratio (INR), prothrombin time (PT), and activated partial thromboplastin time (aPTT). Plasma D-dimer levels were measured using a latex-enhanced photometric immunoassay kit (Siemens, Marburg, Germany) via a CS2500 automatic coagulation analyzer (Sysmex, Kobe, Japan). The values were expressed as ng/mL.

Power analysis was performed using Russ Lenth's power and sample-size calculation application^15^. The primary endpoint of the study was the change in serum D-dimer levels between groups. Our preliminary results showed that mean serum D-dimer levels were 544.95±150.26 in patients diagnosed with HEG and 466.56±101.41 in the healthy pregnancy group. A total of 40 patients in each group were needed with a power of 85%, an α error of 5%, and a β error of 4%. Considering potential dropouts, 100 patients, 50 patients in each group, were included in the study.

Statistical analyses were done with the Statistical Package for the Social Sciences (SPSS 22; IBM SPSS Statistics for Windows, Version 22.0). The normality of variables was tested with the Kolmogorov-Smirnov test. Since the data showed a normal distribution, we compared the demographic characteristics, as well as the biochemical and hematological parameters, between the groups using the independent t-test. The results were presented as mean±standard deviation (SD), and p≤0.05 was considered statistically significant. Pearson's correlation coefficient was used to determine the linear relationship between D-dimer levels and other coagulation parameters among HEG patients.

## RESULTS

A total of 100 pregnant women (n=50 for each group) were included in the study. Missing data from five patients in the HEG group and three patients in the control group were excluded. Consequently, data from 45 patients in the HEG group and 47 patients in the control group were analyzed.

**Figure 1:**
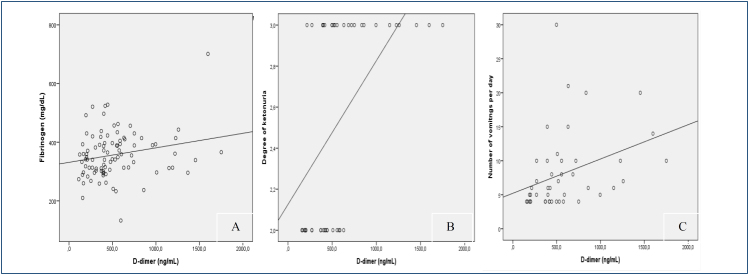
Correlation between serum fibrinogen and D-dimer levels (p=0.039, r=0.215) (A), degree of ketonuria and serum D-dimer levels (p=0.008, r=0.274) (B), number of vomitings per day and serum D-dimer levels (p=0.02, r=0.346) (C).

There were no statistically significant differences between groups in terms of age, week of gestation, and BMI. While 21 of the patients diagnosed with HEG were ketone 2 positive, 24 of them were ketone 3 positive. Serum levels of hemoglobin, white blood cell and platelet counts, PT, aPTT, fibrinogen, D-dimer, and INR were similar between groups (Table 1) (p>0.05). When a D-dimer level of 500 ng/mL was considered a pathological value, 21 (46.7%) patients in the HEG group and 16 (34%) patients in the healthy pregnant group had a D-dimer level above 500 ng/mL (p=0.288). Serum total, low-density lipoprotein (LDL), and high-density lipoprotein (HDL) cholesterol levels were lower in the HEG group than in the control group (p<0.05). No correlations were found between D-dimer levels, maternal age, BMI, and serum biochemical parameters (p>0.05). However, there was a positive association between serum D-dimer levels and serum fibrinogen levels (p=0.039, r=0.215) (Figure 1A). Also, a positive association was observed between serum D-dimer levels with the degree of ketonuria (p=0.008, r=0.274) and the number of vomitings per day (p=0.02, r=0.346) in HEG patients (Table 2, Figures 1B and 1C).

**Table 1:** Comparison of demographic and obstetric characteristics and hematological and coagulation parameters of the groups.

	HEG group (n=45)	Control group (n=47)	p-value
Age (years)	27.16±4.59	27.72±4.83	0.566
Maternal weight (kg)	62.27±13.10	63.00±13.09	0.789
Height (cm)	161.56±4.92	161.09±4.89	0.647
BMI (kg/m^2^)	23.97±4.48	24.40±4.52	0.648
Gravida (n)	2.11±1.54	2.51±1.93	0.277
Abortion (n)	0.33±0.85	0.53±1.10	0.337
Gestational age (weeks)	9.02±1.88	8.46±2.08	0.185
Degree of ketonuria	2.53±0.50	0.00±0.00	<0.001
Number of vomitings per day	7.82±5.93		
Hb (g/dL)	13.47±1.26	13.24±1.39	0.409
WBC (K/μL)	9.03±2.37	9.27±2.43	0.629
PLT (K/μL)	268.31±40.18	291.55±76.75	0.074
PT (s)	15.09±1.41	17.33±19.39	0.441
aPTT (s)	29.40±3.32	28.61±3.04	0.239
INR	1.16±0.11	1.10±0.14	0.064
Fibrinogen (mg/dL)	370.60±84.38	344.55±73.69	0.118
D-dimer (ng/mL)	581.88±387.98	465.23±295.04	0.107
Total cholesterol (mg/dL)	134.64±25.65	152.91±33.64	0.004*
Triglyceride (mg/dL)	85.67±30.11	97.36±38.79	0.111
HDL-cholesterol (mg/dL)	43.11±10.30	49.78±12.21	0.006*
LDL-cholesterol (mg/dL)	86.62±25.22	97.40±25.98	0.047*

Data were presented as mean±SD or number (%),

* p<0.05.

BMI: body mass index; HEG: hyperemesis gravidarum; INR: international normalized ratio; Hb: hemoglobin; PLT: platelet; PT: prothrombin time; aPTT: activated partial thromboplastin time; WBC: white blood cell; SD: standard deviation; HDL: high-density lipoprotein; LDL: low-density lipoprotein.

**Table 2:** Correlation between serum D-dimer levels and other parameters among hyperemesis gravidarum patients.

Variables	R	p-value
Fibrinogen	0.215	0.039*
Hemoglobin	-0.285	0.006*
Degree of ketonuria	0.274	0.008*
Number of vomitings per day	0.209	0.045*
Prothrombin time	-0.116	0.270
Activated partial thromboplastin time	-0.115	0.273
Platelets	-0.042	0.690
International normalized ratio	-0.080	0.451

* p<0.05, r: correlation coefficient.

## DISCUSSION

There is an increasing trend toward using D-dimer testing for the prediction of VTE risk in the general population. This study aimed to evaluate serum D-dimer levels in pregnant women with HEG and investigate their association with other biochemical and clinical parameters. Our findings indicated that serum D-dimer levels, along with other coagulation parameters, did not show significant differences between HEG patients and healthy pregnant controls. However, a positive correlation was observed between serum D-dimer levels and fibrinogen levels, as well as the degree of ketonuria and frequency of vomiting.

Researchers define HEG as one of the conditions in which the oxidant and antioxidant balance in the body is disrupted^3,4^. It was shown that HEG may lead to adverse perinatal outcomes such as preeclampsia, preterm labor, fetal growth restriction, and abortion^16,17^. For this reason, researchers have focused on the etiology and pathogenesis of HEG.

D-dimer is a degradation product of fibrin by plasmin, and it is an indicator of the indirect activation of coagulation and fibrin polymerization^5^. Hansen et al.^18^ showed a significant association between elevated plasma D-dimer levels and an increased risk of incident VTE in the non-pregnant population. Also, in a review analyzing 37 articles, D-dimer elevation was reported to be associated with the risk of first VTE occurrence, VTE recurrence, and mortality with a cutoff value of 500 ng/mL^19^. In the current study, the number of patients with D-dimer concentrations above 500 ng/mL was similar in both groups. Also, serum D-dimer levels were similar between HEG patients and healthy pregnant controls. However, there was a positive correlation between serum D-dimer levels and the degree of ketonuria and frequency of vomiting. Pregnant women tend to have higher D-dimer concentrations as HEG severity increases. However, we failed to assess the cutoff value of D-dimer for the prediction of VTE because there was no case with VTE in the study population.

Increased D-dimer levels have been reported in hospitalized patients who do not have VTE. Many clinical conditions such as infection, inflammation, cancer, trauma, surgery, ischemic heart disease, stroke, and pregnancy lead to an increase in serum D-dimer levels^5-7^. The present study found no significant differences in the mean plasma D-dimer values of HEG patients compared to healthy controls. However, the present study showed a tendency for higher D-dimer concentrations with the severity of HEG. This relationship confirms the hypothesis that HEG is an important risk factor for the thrombotic state. Sadeghi et al.^20^ investigated the reference value for the D-dimer range in the first trimester of pregnancy in diagnosing pulmonary embolism. They reported the cutoff points in the first trimester of pregnancy as 1,701 μg/L. In a systematic review analyzing the results of four studies, it was suggested that D-dimer may be a safe and useful diagnostic tool in the management of pregnant women with suspected VTE^21^. In the current study, all patients had D-dimer levels below 1,701 μg/L, and no patient had clinical findings suggestive of VTE.

This is the first study to evaluate serum D-dimer levels in HEG patients and their associations with other coagulation parameters. The study has several strengths, including its prospective design and well-matched control group. However, it also has limitations, such as the exclusion of patients with comorbidities that could confound the results.

In conclusion, serum D-dimer levels were not different in patients with HEG versus healthy women during the first trimester of pregnancy. Pregnant women tend to have higher D-dimer concentrations as HEG severity increases. Serum D-dimer levels cannot be used for the evaluation of potential VTE in patients diagnosed with HEG. Therefore, in the case of suspected VTE associated with HEG, serum D-dimer ­levels may not be useful for excluding acute VTE. Further studies are necessary to establish a feasible cutoff value for serum D-dimer levels to identify VTE in patients diagnosed with HEG. Clinicians should continue considering a multifactorial approach in assessing thrombotic risk in pregnant women, incorporating both clinical and laboratory data.

## Data Availability

The datasets generated and/or analyzed during the current study are available from the corresponding author upon reasonable request.
